# Transcriptome-wide m^6^A profiling reveals mRNA post-transcriptional modification of boar sperm during cryopreservation

**DOI:** 10.1186/s12864-021-07904-8

**Published:** 2021-08-03

**Authors:** Ziyue Qin, Wencan Wang, Malik Ahsan Ali, Yihan Wang, Yan Zhang, Ming Zhang, Guangbin Zhou, Jian-dong Yang, Changjun Zeng

**Affiliations:** 1 College of Animal Sciences and Technology, Sichuan Agricultural University, 611130 Chengdu, Sichuan China; 2 Farm Animal Genetic Resources Exploration and Innovation Key Laboratory of Sichuan Province, Sichuan Agricultural University, 611130 Chengdu, Sichuan Province China; 3 Department of Theriogenology, Riphah College of Veterinary Sciences, 54000 Lahore, Pakistan

**Keywords:** N^6^-methyladenosine (m^6^A), Boar sperm, Cryopreservation, MeRIP-seq

## Abstract

**Background:**

Cryopreservation induces transcriptomic and epigenetic modifications that strongly impairs sperm quality and function, and thus decrease reproductive performance. N^6^-methyladenosine (m^6^A) RNA methylation varies in response to stress and has been implicated in multiple important biological processes, including post-transcriptional fate of mRNA, metabolism, and apoptosis. This study aimed to explore whether cryopreservation induces m^6^A modification of mRNAs associated with sperm energy metabolism, cryoinjuries, and freezability.

**Results:**

The mRNA and protein expression of m^6^A modification enzymes were significantly dysregulated in sperm after cryopreservation. Furthermore, m^6^A peaks were mainly enriched in coding regions and near stop codons with classical RRACH motifs. The mRNAs containing highly methylated m^6^A peaks (fts vs. fs) were significantly associated with metabolism and gene expression, while the genes with less methylated m^6^A peaks were primarily involved in processes regulating RNA metabolism and transcription. Furthermore, the joint analysis of DMMGs and differentially expressed genes indicated that both of these play a vital role in sperm energy metabolism and apoptosis.

**Conclusions:**

Our study is the first to reveal the dynamic m^6^A modification of mRNAs in boar sperm during cryopreservation. These epigenetic modifications may affect mRNA expression and are closely related to sperm motility, apoptosis, and metabolism, which will provide novel insights into understanding of the cryoinjuries or freezability of boar sperm during cryopreservation.

**Supplementary Information:**

The online version contains supplementary material available at 10.1186/s12864-021-07904-8.

## Background

Cryopreservation is a vital procedure with extensive applications for long-term semen storage, despite the associated risk for sperm cryoinjuries, including impaired motility, fertility, and apoptosis, due to oxidative stress [1–4]. In commercial swine herds, only about 1 % of artificial insemination (AI) worldwide is practiced with post-thawed semen as it results in decreased conception rate and litter size [5–7]. Therefore, the study of molecular markers and underlying mechanisms related to cold shock and freeze tolerance is imperative to improve AI outcomes. Sperm epigenetic markers, such as DNA methylation, histone modification, and non-coding RNAs are associated with successful fertilization and healthier offspring [8, 9]. It is well documented that external chemicals, radiation, and stress can elicit remarkable epigenetic changes in boar sperm. Previously, we have detected dramatic changes in the expression of epigenetic-related genes, such as *Dnmt3a* and *Dnmt3b*, during boar sperm cryopreservation [10]. Likewise, the level of DNA methylation in horse sperm increased significantly after cryopreservation, and this may partially explain the low fertility of mares after insemination with frozen-thawed semen [11]. However, little is known about how RNA methylation modifications in sperm respond to external stimuli.

So far, over 100 chemical modifications on RNAs, especially on transfer RNA (tRNA) and ribosomal RNA (rRNA) have been discovered [12, 13]. N^6^-methyladenosine (m^6^A) is the most prevalent post-transcriptional modification of mRNA and is ubiquitous in various species, including mammals [14, 15], plants [16], fruit flies [17], yeast [18], and viruses [19]. The mammalian m^6^A methylation is catalyzed by several methyltransferases, including methyltransferase-like 3 (METTL3), methyltransferase-like 14 (METTL14), and Wilms’ tumor 1-associating protein (WTAP). Conversely, Fat mass and obesity-associated protein (FTO) and alkB homolog 5 (ALKBH5) act as demethylases and remove the m^6^A modification from RNAs. YT521-B homology (YTH) domain family proteins (YTHDF1/2/3), a class of specific m^6^A reader proteins, are responsible for the biological functions of m^6^A modification [20]. In addition, m^6^A modifications are an extremely important epigenetic modification that regulate post-transcriptional gene expression by regulating the splicing, stability, degradation, and translation of m^6^A modified mRNAs [21–23].

In mammals, increasing evidence supports the crucial biological functions of mRNA m^6^A methylation in sperm. Previous studies indicated that knock-out of m^6^A modification enzymes leads to lower sperm count, motility, and fertility in mouse [24, 25]. Tang et al. found that m^6^A modification controls the fate of mRNAs with a long 3’UTR by ALKBH5-dependent demethylation during spermatogenesis [26]. Further work showed that depletion of ALKBH5 facilitates the biogenesis of translatable circRNAs, whose translation initiation is mediated by YTHDF3-dependent recognition of start codon by m^6^A methylation [27]. Compared with normal sperm, the m^6^A content and *METTL3* expression in asthenospermic patients were significantly increased, suggesting that an abnormal increase of m^6^A modification in human sperm may greatly affect sperm motility and m^6^A modification enzymes (*METTL3*, *METTL14*, *FTO*, *ALKBH5*, *YTHDF2*) were implicated in modulating m^6^A contents in sperm RNA [28]. Landfors et al. identified two missense mutations of the FTO protein in human sperm, which led to functional defects in m^6^A demethylation and declining sperm quality [29]. Generally, the extent of m^6^A methylation, as well as the m^6^A-protein level, may reflect quality and fertilizing capacity of sperm during cryopreservation.

RNA-seq is a widely used technology for high-throughput analysis of transcriptomic profiles, and the study of the sperm transcriptomes is crucial for understanding its biology and role in fertility [30]. Multiple studies have used RNA-seq to evaluate differentially expressed transcripts in boar sperm and have demonstrated the involvement of multiple genes in regulating vital physiological functions  [31, 32]. Moreover, these transcripts can be used as a reference for the identification of markers of sperm quality in pigs. In addition, methylated RNA immunoprecipitation sequencing (MeRIP-seq) is a common approach based on RNA-seq to profile and predict m^6^A modifications across the transcriptome. MeRIP-seq can be employed to detect m^6^A modifications at specific loci to provide insight into the regulatory mechanisms underlying environmental response [33]. In this study, fresh sperm (Fs) and frozen-thawed sperm (Fts) from boar were used for profiling the transcriptome-wide m^6^A methylation patterns by MeRIP-sEq. In addition, we performed RNA-Seq and carried out a combined analysis of m^6^A methylation and mRNA levels. We found highly diverse m^6^A patterns between Fs and Fts, and speculate that study of m^6^A modification patterns may present an opportunity to deepen our understanding of the role of epigenetic modifications in regulating sperm function during cryopreservation.

## Results

### Cryopreservation of boar sperm altered mRNA and expression of m^6^A modification enzymes

**Fig. 1:**
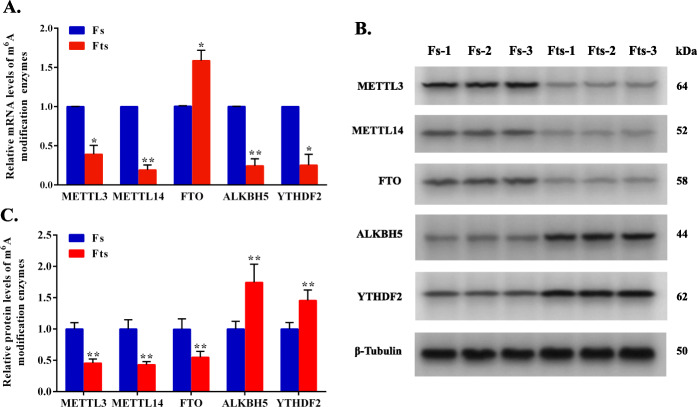
m^6^A modification enzymes of boar sperm are dysregulated after cryopreservation. **A** Relative mRNA expression levels of m^6^A modification enzymes in Fs and Fts assessed by RT-qPCR. **B** WB bands illustrating the protein levels of m^6^A modification enzymes and β-tubulin in Fs and Fts. **C** Histogram showing the relative protein levels of m^6^A modification enzymes in Fs and Fts. Values were normalized using β-tubulin protein as an internal reference. Fs, fresh sperm; Fts, frozen-thawed sperm. “*P < 0.05, **P < 0.01”

Using RT-qPCR and western blot (WB), we assessed differences in mRNA and protein levels of five major enzymes responsible for m^6^A modification between Fs and Fts, including METTL3, METTL14, FTO, ALKBH5 and YTHDF2. The mRNA expression levels of *METTL3*, *METTL14*, *ALKBH5*, and *YTHDF2* were significantly decreased in the Fts group, whereas the *FTO* gene showed significantly increased expression (*P* < 0.05) (Fig. 1A). The protein levels of METTL3, METTL14, and FTO were downregulated in response to cryopreservation (*P* < 0.01), whereas ALKBH5 and YTHDF2 were upregulated in the Fts group (*P* < 0.01, Fig. 1B C). Therefore, we speculate that cryopreservation induces m^6^A methylation by regulating the mRNA and protein levels of five major enzymes responsible for m^6^A modifications.

### Overview of transcriptome-wide m^6^A methylation of boar Fs and Fts

An average of more than 78,000,000 clean reads were obtained from 12 libraries in Fs IP and Fts IP groups by MeRIP-Seq (Table S1). Furthermore, the mapping ratio of clean reads in both Fs IP and Fts IP groups exceeded 75.09 % when matched to the reference genome (UCSC susScr11) (Table S1).

**Fig. 2:**
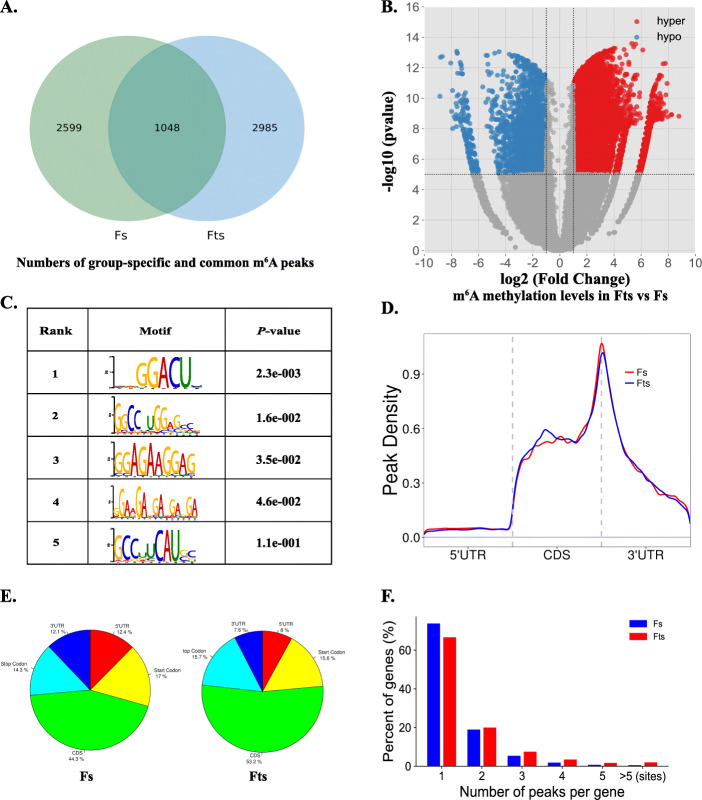
Overview of m^6^A methylation in Fs and Fts. **A** Venn diagram of common m^6^A peaks within mRNAs in Fs and Fts. **B** Volcano plots of all distinct m^6^A peaks (|fold change| ≥ 2 and *P* < 0.00001). hyper: hyper-methylated; hypo: hypo-methylated. **C** The top five motif sequences for m^6^A-containing peaks from all samples. **D** Proportion of genes harboring different numbers of m^6^A peaks in Fs and Fts. **E** Pie charts showing the percentage of DMMSs in different gene contexts. **F** Enrichment of m^6^A peaks along transcripts. Each transcript is divided into three segments, including 5’UTR, CDS, and 3’ UTR. Fs, fresh sperm; Fts, frozen-thawed sperm

A total of 3,647 and 4,033 m^6^A peaks were identified in Fs and Fts, respectively. Of these, 1,048 peaks (~ 16 % of all peaks in both Fs and Fts) were common to both Fs and Fts samples (Fig. 2A). Compared with Fs, 1,613 significantly hyper-methylated peaks among 1,442 mRNAs and 315 significantly hypo-methylated peaks within 312 mRNAs (|fold change| ≥ 2 and *P* < 0.00001) in Fts were identified (Fig. 2B, Table S2-1, S2-2). The top 10 significantly increased and decreased m^6^A peaks in Fts are listed in Table S3 and Table S4, respectively.

To determine the conserved RRACH (R = purine, A = m^6^A and H = non-guanine base) motif in all identified m^6^A peaks, the top 2000 m^6^A-containing peaks from all sperm samples were analyzed. Out of these, five motif consensus sequences were listed by enrichment, consistent with former studies, reinforcing the authenticity of the data (Fig. 2C). In addition, metagene profile analysis indicated that m^6^A peaks were preferentially located at coding regions (CDS), at start codons, and near stop codons in both Fs and Fts groups; Fts had higher proportion of m^6^A peak enrichment in CDS than Fs (Fig. 2D and E). Generally, about 80 and 70 % of m^6^A-containing genes had only one m^6^A peak in Fs and Fts, respectively. The number of transcripts containing two or more peaks in Fts was greater than in Fs (Fig. 2F).

### Distribution of differentially methylated m^6^A sites (DMMSs)

**Fig. 3:**
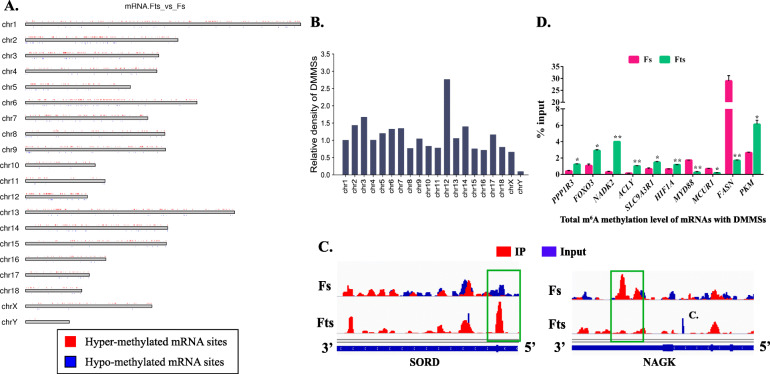
Distribution of differentially methylated m^6^A sites (DMMSs). **A** Chromosomal distribution of all DMMSs within mRNAs. **B** Relative occupancy of DMMSs in each chromosome. **C** Data visualization analysis of two representative hyper- and hypo-methylated genes in Fts and Fs groups. The green rectangles show the locations of differentially methylated peaks. **D **The validation of total m^6^A methylation level of ten mRNAs with differentially methylated sites selected from MeRIP-seq data. Fs, fresh sperm; Fts, frozen-thawed sperm. “*P < 0.05, **P < 0.01”

All differentially methylated m^6^A sites (DMMSs) within mRNAs were mapped to chromosomes to evaluate their distribution profiles (Fig. 3A). The top five chromosomes with the highest relative densities of DMMS normalized to chromosome length, in decreasing order, were 12, 3, 14, 2 and 7 (Fig. 3B). By using Integrative Genomics Viewer (IGV, v2.8.2) software, we displayed two representative DMMSs selected from MeRIP-seq, *SORD* (hypermethylated peak) and *NAGK* (hypomethylated peak), which showed altered m^6^A intensity (Fig. 3C). Further, we conducted an independent methylated RNA immunoprecipitation-qPCR (MeRIP-qPCR) experiment to detect total mRNA m^6^A levels of ten randomly selected genes with DMMSs. Notably, six hypermethylated genes, *FOXO3*, *NADK2*, *ACLY*, *HIF1A*, *SLC9A3R1*, and *PKM*, and one hypomethylated gene, *FASN*, were reported to be involved in regulation of sperm quality. All of these genes have significantly altered total mRNA m^6^A levels as measured by MeRIP-qPCR (Fig. 3D). Among these, the m^6^A levels of nine genes were consistent with MeRIP-seq data; conversely, the total m^6^A level of *FASN* was reduced in Fts by MeRIP-qPCR but showed an increase in the MeRIP-seq data. These results confirm the accuracy of our sequencing data.

### GO and KEGG pathway analysis of genes containing significantly altered m^6^A peaks (DMMGs)

**Fig. 4:**
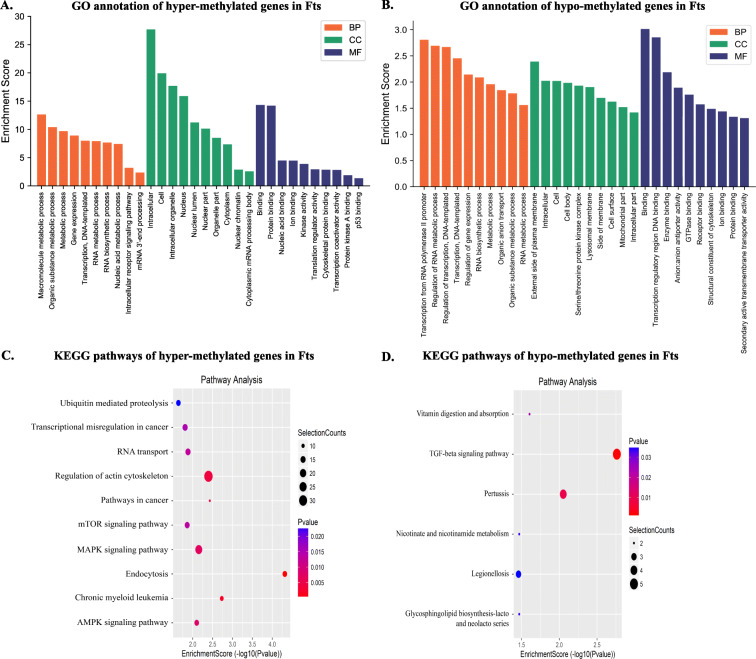
GO and KEGG analyses of coding genes containing altered m^6^A peaks in Fs and Fts. **A** Distribution of GO terms significantly enriched for hyper-methylated genes in Fts. **B** Distribution of GO terms significantly enriched for the hypo-methylated genes in Fts. **C** Significantly enriched pathways for the hyper-methylated genes in Fts. **D** Significantly enriched pathways for the hypo-methylated genes in Fts. Fs, fresh sperm; Fts, frozen-thawed sperm

In order to explore the physiological functions of m^6^A methylation in Fts, the genes containing significantly altered m^6^A peaks (differentially methylated m^6^A genes, DMMGs) were subjected to GO and KEGG pathway analysis. GO analysis (biological process, BP; cellular component, CC; molecular function, MF; Table S5-1, S5-2) of the hyper- and hypo-methylated genes in Fts showed that hyper-methylated genes were significantly (*P* < 0.05) involved in metabolism, including in macromolecule metabolic process and organic substance metabolic process, gene expression, and transcription (ontology: BP). Furthermore, these genes also play a part in cell and intracellular organelles (ontology: CC) and protein binding (ontology: MF, Fig. 4A). Hypo-methylated genes in Fts were significantly enriched in transcription from RNA polymerase II promoter, regulation of RNA metabolic process, regulation of transcription, and DNA-templating (ontology: BP), external side of plasma membrane (ontology: CC), and transcriptional regulatory region DNA binding and enzyme binding (ontology: MF, Fig. 4B). Moreover, KEGG pathway analysis revealed that hyper- and hypo-methylated m^6^A sites representing genes in Fts were enriched in mTOR, AMPK, MAPK, and TGF-beta signaling pathways (Fig. 4C, D, Table S6-1, S6-2).

### Joint analysis of DMMGs and differentially expressed genes (DEGs)

**Fig. 5:**
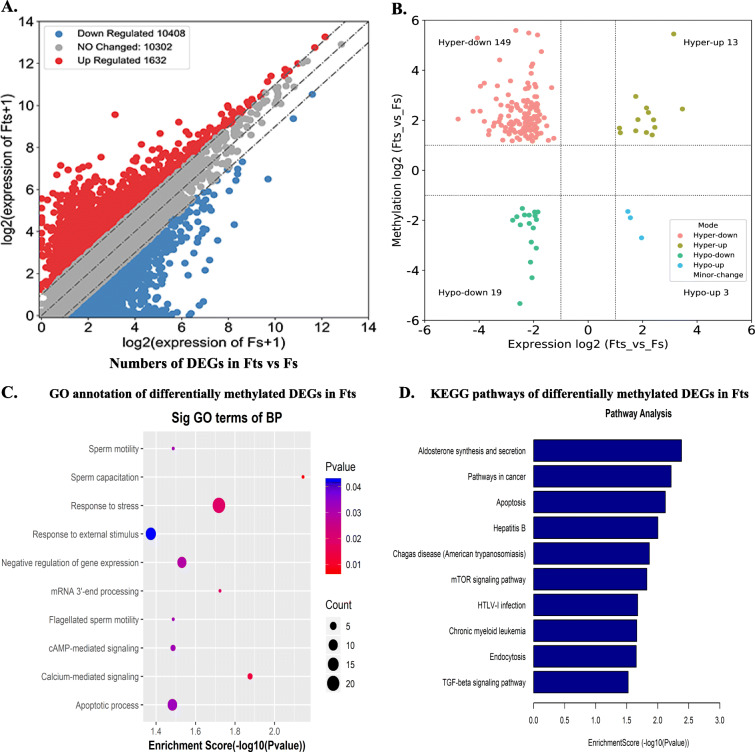
Joint analysis of DMMGs and DEGs. **A** Scatter plots showing the DEGs between the Fs and Fts (|fold changes| ≥ 2 and *P* < 0.05). **B** Four quadrant graphs exhibiting the DEGs containing DMMSs. **C** The major GO terms of significantly enriched differentially methylated DEGs by GO analysis. **D** The top ten pathways of significantly enriched differentially methylated DEGs by KEGG analysis. Fs, fresh sperm; Fts, frozen-thawed sperm

Given the indispensable function of RNA m^6^A modifications in regulating gene expression, transcriptome profiles of altered genes in Fts were determined by RNA-sEq. Scatter plots showed the presence of 295 significantly up-regulated genes and 2,071 significantly down-regulated genes (|fold change| ≥ 2, *P* < 0.05) between Fs and Fts (Fig. 5A, Table S7-1, S7-2). All genes were divided into four groups based on joint analysis of DMMGs and DEGs, including 13 hyper-methylated & up-regulated genes (hyper-up), 19 hypo-methylated & down-regulated genes (hypo-down), 149 hyper-methylated & down-regulated genes (hyper-down), and 3 hypo-methylated & up-regulated genes (hypo-up) (Fig. 5B). Notably, 88.7 % (149/168) of the down-regulated mRNAs contained hyper-methylated m^6^A peaks. GO and KEGG analysis revealed that DEGs containing hyper- and hypo- methylated m^6^A peaks were significantly enriched in many important biological processes and pathways, such as sperm capacitation, calcium-mediated signaling, sperm motility, and apoptosis (Fig. 5C, D, Table S8). Nine differentially methylated DEGs between Fs and Fts, associated with sperm quality and function, including regulation of sperm motility and capacitation, are listed in Table 1.

**Table 1:** Functions of the differentially expressed and methylated DEGs between Fs and Fts during cryopreservation

Gene symbol	Regulation (Fts vs. Fs)						Description
	**Methylation**	**Fold Change**	***p*_value**	**mRNA expression**	**Fold Change**	***p*_value**	
*ACLY*	Up	2.8	5.18E-06	Down	-inf	2.72e-02	Regulation of the ram sperm energy metabolism and ATP supplement [34, 35]
*PDE4A*	Up	2.8	4.67E-06	Up	2.3	3.14e-02	Regulation of bovine sperm motility [36]
*NFATC3*	Up	11.3	3.20E-06	Down	-inf	7.20e-03	Differentially represented between fresh- and frozen-sperm in boar [31]
*BCL2L1*	Up	3.1	8.07E-06	Down	-inf	2.80e-03	Cell apoptosis protecting [37]
*CARD6*	Up	2.9	4.07E-06	Down	-inf	5.00e-05	Regulation of apoptosis [38]
*PIK3R1*	Up	3.2	2.56E-06	Down	-inf	5.00e-05	Involved in actin polymerization during bovine sperm capacitation [39]
*NCALD*	Up	2.3	1.13E-06	Down	-inf	5.00e-05	Regulation of Calcium-modulated transduction in human and bovine sperm [40]
*SOX9*	Up	11.0	8.73E-07	Down	-inf	5.00e-05	Regulate male fertility in mice [41]
*PLCB1*	Up	7.7	5.65E-06	Down	-inf	9.50e-04	Essential for acrosome reaction and fertilization in mouse sperm [42]

Note: -inf means negative infinity

## Discussion

The process of freezing-thawing induces dramatic changes in sperm, including osmolarity, volume, and oxidative stress [43, 44]. These rapid transitions could affect cell membrane fluidity, plasma membrane integrity, and DNA structure [45, 46]. In addition, dysregulation of redox homeostasis [47] and mitochondrial activity [48], and expression of cryoinjury-related genes [49, 50] could eventually impair morphology, motility, and metabolism of frozen-thawed sperm [51]. Recent studies indicated that cryopreservation as an environmental stimulus alters transcript, non-coding RNA (miRNAs, LncRNAs etc.), and protein levels. These altered transcripts, non-coding RNA, and proteins were demonstrated to be associated with post-thawed sperm quality, such as motility, survival, fertility, and early embryonic development [52]. Moreover, the process of freezing and thawing also induces an alteration of sperm epigenetics [10, 11]. However, transcriptome-wide epigenetic modifications in sperm have not been reported. Previous studies demonstrated that m^6^A methylation was significantly associated with cellular response to environmental stimuli, such as endocrine-disrupting chemicals [53], oxidative stress [54], and inflammation [55]. In this study, we first found that the methyltransferases METTL3 and METTL14 were down-regulated in frozen-thawed sperm. Conversely, the protein levels of FTO, ALKBH5, and YTHDF2 were inconsistent with their mRNA levels. Previous studies demonstrated that freezing-thawing treatment affects mRNA-protein interactions and makes mRNA more susceptible to degradation [56]. Moreover, the decrease of some transcripts might also result from an increase in translation for more protein synthesis [57]. Oxidative stress could induce elevated m^6^A levels and up-regulate the expression of *FTO* without affecting protein levels. Thus, cryoprotectant might exert a protective effect against oxidative stress by inhibiting of the expression of FTO in sperm [58]. mRNA levels of key m^6^A modification enzymes, including METTL3, METTL14, FTO, and YTHDF2, are highly correlated with the proportion of m^6^A modifications of total mRNA [59]. Germ cell-specific inactivation of METTL3 and METTL14 causes loss of m^6^A modifications [24]. Overexpression of ALKBH5 can suppress the expression of m^6^A-modified mRNA [60]. Additionally, the m^6^A modification reader YTHDF2 shows identical binding to all m^6^A sites in mRNAs and mediates the degradation of m^6^A-mRNAs [61, 62]. These findings suggest that cryopreservation may affect the methylation level and stability of m^6^A-modified mRNA, which reflects the metabolic status of a cell under environment stress. In the present study, we investigated the importance of m^6^A methylation in sperm by using MeRIP-seq to determine the mRNA m^6^A profiles of Fs and Fts.

We discovered unique patterns of m^6^A modifications in mRNA from Fs and Fts. The motifs of m^6^A modification sites in sperm mRNA were revealed by enrichment analysis, which showed that the RRACH consensus sequences were consistent with other studies in various species [14, 63, 64]. Further, we found that m^6^A peaks were especially enriched in CDS, at start codons, and near stop codons, which is consistent with prior work in human and mice [14, 65]. Moreover, m^6^A methylation patterns in sperm are the same as in other tissues of the pig, such as fat and muscle [66], which further confirms that features of RNA m^6^A methylation are conserved in pig. We identified numerous m^6^A peaks that varied widely among individual transcripts, and most of them harbored only one or two m^6^A peaks, which was consistent with previous studies in humans [67] and chickens [68]. Analysis at the whole chromosome level showed that mRNAs containing altered m^6^A peaks were transcribed from all chromosomes, especially autosomes [67, 69], indicating that m^6^A modification patterns in Fts are widely changed and may involve many pathways that influence the function of sperm. Furthermore, the total m^6^A level of nine transcripts (*PPP1R3*, *FOXO3*, *NADK2*, *ACLY*, *SLC9A3R1*, *HIF1A*, *MYD88*, *MCUR1*, and *PKM*) were evaluated using MeRIP-qPCR and found to be consistent with the MeRIP-seq results. The findings suggest that transcript identification and estimates of expression were highly reliable.

It has been demonstrated that a variety of signaling pathways and regulatory mechanisms participate in normal sperm function. Herein, GO and KEGG pathway analyses were performed to deduce potential functions of DMMGs. GO analysis revealed that coding genes with altered m^6^A peaks were mainly involved in biological processes such as metabolism, gene expression, and regulation of RNA metabolism. Cryopreservation impairs numerous proteins implicated in mitochondrial tricarboxylic acid (TCA) cycle and oxidative phosphorylation, which contribute to proper metabolism and oxidoreductase activity in sperm [70]. Cryodamage is also involved in the degradation of certain mRNAs; therefore, it could impair the function of relevant proteins [50]. Natural modifications of cellular RNA enable the orderly metabolism and function of diverse RNA species, thereby affecting gene expression [71]. Recent studies have suggested that environmental perturbations could induce epigenetic changes in the testis. After exposure to environmental toxins, genes with differentially methylated DNA regions or differentially expressed mRNA in Sertoli cells of F3 generation rats were enriched in categories including metabolism, transcription, and cytoskeleton [72]. Therefore, m^6^A methylation may mediate the regulation of sperm RNA levels and metabolism during cryopreservation. KEGG analysis revealed that DMMGs were significantly enriched in the ubiquitin mediated proteolysis, AMPK, mTOR, and MAPK signaling pathways, which are known to control stress response, apoptosis, and capacitation in sperm [73, 74]. Ubiquitin acts as a molecular marker and tags proteins for degradation by the proteasome. High sperm surface ubiquitination is coupled with lower developmental competence of pig embryos [75]. Cryopreservation induced a significant decrease in the percentage of ubiquitinated boar sperm, and the ability of frozen-thawed sperm to capacitate and of the acrosome to react were both influenced by ubiquitination [76]. AMPK is a key kinase involved in regulating the cellular redox state by switching metabolic pathways under stressful conditions. Zhu et al. reported that resveratrol is beneficial for improving the quality of post-thaw boar sperm by activating the AMPK pathway to reduce sperm apoptosis [3]. The PI3K/AKT/mTOR signaling pathway is involved in the regulation of sperm autophagy and mTOR is considered a central integrator of several signals, allowing it to regulate metabolism and redox balance [77]. Furthermore, the process of freezing and thawing can activate p53 via the p38/MAPK pathway and subsequently cause apoptosis in sperm [78]. Similar KEGG pathways, including MAPK, TGF-beta, and ubiquitin-mediated proteolysis signaling pathways, which are directly linked with sperm quality, contain miRNAs that were differentially expressed in sperm exposed to fluoride [79]. In addition, a series of DMMGs were found to participate in stress response, apoptosis, substrate metabolism, and sperm motility. Sirtuin 1 (*SIRT1*), a histone deacetylase, was found to be involved in regulating transcription, energy metabolism, and oxidative stress and to contribute to sperm abnormalities in infertile men [80–82]. Evidence indicates that increased expression of *SIRT1* could suppress spermatogonial cell apoptosis, and DNA hyper-methylation of *SIRT1* promotes down-regulation of *SIRT1* expression [83, 84]. Defects in Parkinsonism-associated protein 7 (*Park7*, also known as *DJ-1*), an oxidative stress response product, have been widely reported to be correlated with male infertility [85–87]. Recently, *Park7* was observed as a target gene for the mitochondria-related miRNA miR-4485-3p which may link mitochondrial dysfunction and male asthenozoospermia [88]. Kinesins (*KIFs*) are a superfamily of motor proteins and have been reported to play a role in sperm motility and apoptosis [89–92]. In our data, some differentially methylated *KIF* genes were also detected, including *KIF1B*, *KIF5A*, *KIF5B*, and *KIF15*. Interestingly, both hyper- and hypo-methylated peaks on the mRNA of *KIF5B* (2.8- and 11.6-fold change, respectively) were detected. Similarly, *NDUFS1*, an oxidative phosphorylation marker, whose mRNA methylation increased 3.2-fold after cryopreservation in our study, was detected at low levels in semen collected from patients with testicular cancer and at high levels in the sperm of infertile men [93, 94]. Sorbitol dehydrogenase (*SORD*) converts sorbitol to fructose, which can be further metabolized via the glycolytic pathway to yield ATP [95]. This reproduction-related gene is also involved in the maturation and capacitation of sperm [96]. Moreover, N-acetyl glucosamine kinase (*NAGK*) was predicted to significantly affect ATP production and regulate metabolism in asthenozoospermia [97]. As shown in Fig. 3C, *SORD* and *NAGK* mRNA were hyper-methylated and hypo-methylated in the Fts group, respectively. In the present study, all of the above-mentioned genes in sperm showed different m^6^A methylation after cryopreservation, but their expression levels did not change significantly. Thus, we propose that m^6^A methylation may affect other conditions related to these genes to regulate the viability, motility, and energy metabolism of boar sperm, which is should be investigated in future studies.

Our previous data from transcriptome sequencing of fresh and frozen-thawed boar sperm revealed that DEGs in frozen sperm are enriched in many important biological processes and pathways, such as sperm motility, metabolism, and apoptosis [98], which is in accordance with our present study of differentially methylated DEGs showing enrichment in these pathways (Table 1). Among these genes, ATP citrate lyase (*ACLY*), a vital gene involved in the TCA cycle whose mRNA was hyper-methylated and down-regulated in the Fts group, has been implicated in male infertility [99]. Further, previous studies also demonstrated that *ACLY* can regulate sperm energy metabolism and ATP production [34, 35]. Phosphodiesterase 4 A (*PDE4A*), whose mRNA was found to be hyper-methylated and up-regulated after cryopreservation in our study, regulates the motility of bovine sperm [36]. In addition, studies have shown that *PDE4A* can modulate sperm chemotaxis by controlling the level of cAMP during fertilization [100, 101]. The mRNA of nuclear factor of activated T cells 3 (*NFATC3*), a potential marker for predicting the freezability of boar sperm [31], was found to be hyper-methylated and down-regulated in the Fts group. Moreover, BCL2-like 1 (*BCL2L1*), an apoptosis-related gene was reported to prevent apoptosis by inhibiting the release of cytochrome c from mitochondria [37]. Claudin domain containing 1 (*CLDND1*) and Caspase recruitment domain family member 6 (*CARD6*) were found to regulate the apoptosis in breast cancer and non-alcoholic fatty liver disease, respectively [38, 102]. In our combined analysis, we found simultaneous changes in the mRNA m^6^A methylation and expression levels of these genes in the Fts group. Numerous studies have substantiated that m^6^A modification in mRNAs could control RNA translation and transcript fate [103, 104]. The impacts of m^6^A on the transcriptome are attributed to the cross-talk among m^6^A readers, writer-complex components, as well as potential erasers, the significance of which remains to be elucidated. Liu et al. reported that knockout of METTL3 or YTHDC1 could enhance chromatin accessibility and activate transcription in an m^6^A-dependent manner [105]. Additionally, knock-down of the m^6^A reader HNRNPA2B1 in human esophageal epithelial cells decreased mRNA expression of *ACLY* [106], and depletion of FTO leads to increased m^6^A levels in total RNA and reduces protein levels of ACLY in HepG2 cells [107]. These results indicate that m^6^A modification can modulate ACLY expression; therefore, it will be worthwhile to further explore the correlation between ACLY activity and m^6^A levels, as well as the exact mechanism underlying ACLY regulatory effects on sperm.

## Conclusions

Our study, for the first time, comprehensively characterized transcriptome-wide m^6^A methylation profiles in boar spermatozoa. We revealed general characteristics, topological patterns, and differences of m^6^A modification and methylation profiles between fresh and frozen-thawed boar sperm. Compared to controls, the number and enrichment levels of m^6^A modified sites on transcripts, as well as the percentage of m^6^A peaks distributed among coding regions, were increased in frozen-thawed sperm. Moreover, joint analysis of MeRIP-seq and RNA-seq data indicated that DEGs containing DMMSs participate in sperm metabolism, apoptosis, and motility. The process of cryopreservation dysregulates m^6^A modification of mRNA, which may be responsible for cryoinjuries or freezability of boar sperm. Together, our work provides new evidence that cryopreservation induces epigenetic modifications of sperm. However, further studies should be conducted to better elucidate the correlation between m^6^A mRNA modification and sperm function.

## Methods

### Animal ethics statement

All procedures for animal treatments were reviewed and written approved by the Institutional Animal Care and Use Committee in the College of Animal Science and Technology, Sichuan Agricultural University, Sichuan, China, under permit No. DKYB20081003.

### Semen collection and cryopreservation

Fresh ejaculates with sperm-rich fractions were collected from three healthy, mature, and fertile Duroc boars, provided by Pengzhou Jinzhu Agricultural Development Co., Ltd (Pengzhou, Sichuan, China), using the gloved-hand technique during the autumn-winter period. All ejaculates with morphologically normal, more than 0.8 sperm motility and 1 × 10^8^ mL^− 1^ of sperm concentration were used. The basic extender used for sperm dilution was Beltsville thawing solution (BTS). The fresh ejaculates of each boar were equally divided into two parts and one was immediately exposed to liquid nitrogen (− 196 °C) and then stored at -80 °C for RNA extraction (Fs). Another part (Fts) was cryopreserved according to our previously described procedure [108]. Briefly, all semen samples were the diluted and slowly cooled to 17 °C for 2 h, then centrifuged for 5 min at 1800 rpm. The sperm pellets were diluted with lactose-egg yolk (LEY) extender containing 11 % β-lactose (w/v) and 20 % hen’s egg yolk (v/v) and slowly cooled to 4 °C for 4 h. Then, the semen samples were further diluted (2:1) with a second freezing extender (LEY supplemented with 6 % glycerol) and equilibrated at 4 °C for 30 min. Subsequently, the semen was loaded in previously labeled 0.2 mL straws (FHK, Tokyo, Japan) and equilibrated at approximately − 130 °C above liquid nitrogen vapor for 15 min, then the straws were plunged in liquid nitrogen (-196 °C) until use.

### Total RNA extraction, cDNA synthesis and RT-qPCR

Before total RNA extraction, seminal plasma was removed by centrifuged for 5 min at 4000 rpm and washed with phosphate buffer solution (PBS) for three times. To eliminate somatic cell contamination, sperm pellet was treated with 0.5 % Triton X-100 (Coolaber, Beijing, China) [109]. Then, Total RNA from boar sperm was extracted with Trizol LS Reagent (Invitrogen Corporation, Carlsbad, CA, USA) according to the manufacturer’s instructions. Furthermore, the concentration and purity of sperm RNA were determined by a Nanodrop (Thermo Fisher Scientific, Wilmington, DE, USA), while Agilent 2100 Bioanalyzer (Agilent Technologies, Santa Clara, CA, USA) was used to measure the RNA integrity. The SYBR Premix Ex Taq II Reagent Kit (Takara Biotech, Dalian, China) was used for RT-qPCR on the CFX 96 Real-Time PCR Detection System (Bio-Rad, Hercules, CA, USA). Briefly, 5 µL SYBR Green I Premix, 0.5 µL each of forward and reverse primers, 1 µL of cDNA, and sufficient RNase-free water were mixed to reach a total volume of 10 µL. Then, the mixture was subjected to thermal cycling as follows: an initial denaturation step at 95 °C for 3 min, 40 cycles of amplification at 95 °C for 5 s, and primer-specific annealing temperatures were applied for 30 s. Relative expression levels were determined using the 2^−∆∆CT^ method [110]. According to counterparts in GenBank, all the primers were designed using NCBI Primer-Blast search (Table S9). The housekeeping gene, *GAPDH*, was used as the reference to evaluate the relative expression level of mRNAs [111].

### Western blot analysis

Western blot of METTL3, METTL14, FTO, ALKBH5 and YTHDF2 was performed according to previous report [109] with some modifications. Briefly, after seminal plasma was removed, sperm pellets were resuspended in a RIPA buffer (containing 1 % phenylmethylsulfonyl fluoride), placed on ice for 30 min and then centrifuged at 12,000 rpm, 4 °C, for 5 min. The concentration of total protein was measured with a bicinchoninic acid (BCA) kit (Solarbio, Beijing, China) according to the manufacturer’s protocol. The protein was separated by 12 % SDS-PAGE and electrophoretically transferred to PVDF membrane (Beyotime, Shanghai, China). Non-specific binding sites of protein were blocked in QuickBlock Western Buffer (Beyotime, Shanghai, China) for 1 h at room temperature and then incubated with primary antibodies [Anti-METTL3 (ab195352, Abcam), Anti-METTL14 (PA5-43606, Thermo Fisher), Anti-FTO (ab94482, Abcam), Anti-ALKBH5 (ab195377, Abcam), Anti-YTHDF2 (24744-1-AP, Proteintech), and anti-β-tubulin (ab21058, Abcam)] diluted in 5 % BSA in TBST (METTL3: 1:1000, METTL14: 1 µg/ml, FTO: 1 µg/ml, ALKBH5: 1:1000, YTHDF2: 1:5000, and β-tubulin 1:1000) overnight at 4 °C followed by incubation with HRP-conjugated secondary antibodies [goat anti-Rabbit IgG H&L (ab6721, Abcam), 1:10,000 dilution]. After washing the membrane with TBST (Beyotime, Shanghai, China), enhanced chemiluminescence detection was performed by using Immun-Star™ WesternC™ Chemiluminescence Kit (BIO-RAD, Hercules, CA, USA) according to the manufacturer’s protocol. The development of PVDF membrane were performed using ChemiScope 6000 Exp (CLiNX, Shanghai, China). Subsequently, band intensities were analyzed using a Gel-Pro Analyzer (Media Cybernetics, Bethesda, MD, USA). β-tubulin was used as the reference protein.

### High-throughput m^6^A and RNA sequencing with data analysis

High-throughput m^6^A services were provided by Cloudseq Biotech Inc. (Cloudseq, Shanghai, China). Briefly, total RNA was extracted using Trizol LS Reagent (Invitrogen Corporation, Carlsbad, CA, USA) following the manufacturer’s instructions. The Ribo-Zero rRNA Removal Kit (Illumina, Inc., San Diego, CA, USA) was used to reduce the ribosomal RNA content. Then, the RNA was chemically fragmented into fragments about 100 nucleotides in length using fragmentation buffer (Illumina, Inc., CA, USA). RNA fragments were incubated with anti-m^6^A polyclonal antibody (Synaptic Systems, Göttingen, Germany) in immunoprecipitation (IP) buffer for 2 h at 4°C. The mixture was then immunoprecipitated by incubation with protein-A beads (Thermo Fisher Scientific, Waltham, MA, USA) for 2 h at 4°C. Then, bound RNA was eluted from the beads with N^6^-methyladenosine (BERRY & ASSOCIATES, Ann Arbor, MI, USA) in IP buffer and then extracted with Trizol Reagent. The NEBNext® Ultra II Directional RNA Library Prep kit (New England Biolabs, Ipswich, MA, USA) was used to construct RNA sequence libraries for the non-immunoprecipitated input RNA samples (mRNA-seq) and immunoprecipitated IP RNA samples (MeRIP-seq). Library quality control was performed using the BioAnalyzer 2100 (Agilent, Santa Clara, CA, USA) and high-throughput sequencing was carried out in 150-bp double-end mode on an Illumina HiSeq 4000 sequencer (Illumina, Inc., San Diego, CA, USA). Image analysis, base recognition, quality control and original reads (Raw Data) were generated with the Illumina HiSeq 4000 sequencer. First, quality control was performed by Q30, which was followed by trimming of the 3’ adaptors and removal of low-quality reads using cutadapt software (v1.9.3) [112]. Second, the clean reads of all samples were matched to the reference genome (UCSCsusScr11, http://hgdownload.soe.ucsc.edu/goldenPath/susScr11/bigZips/susScr11.fa.gz) using Hisat2 software (v2.0.4) [113]. Third, the Model-based Analysis of ChIP-Seq (MACS) software [114] was used to identify the methylated genes in each sample, and non-immunoprecipitated input RNA was used as a correction for MACS peak calling. Differentially methylated sites with a |fold change| ≥ 2 and *P* < 0.00001 were identified with the diffReps software [115], and a proprietary program was used to screen the peaks on the mRNA for corresponding annotation. Identified m^6^A peaks were subjected to motif enrichment analysis using STREME software (v5.3.0) [116]. For RNA sequencing, raw counts of each feature were harvested by HTseq (v0.9.1) [117], and differentially expressed genes were identified using EdgeR software (v3.16.5) [118] with a |fold change| ≥ 2 and *P* < 0.05. GO analysis and KEGG pathway enrichment analysis were performed on the differentially methylated protein coding genes using the GO (www.geneontology.org) and KEGG (www.genome.jp/kegg) databases.

### MeRIP-qPCR

To validate MeRIP-seq results, the Magna MeRIP m^6^A kit (Millipore, Billerica, MA, USA) was used according to the manufacturer’s instructions. Briefly, poly(A) RNA was first purified from 50 µg of total RNA using the Dynabeads™ mRNA Purification kit (Invitrogen Corporation, Carlsbad, CA, USA) and one-tenth of the RNA was saved as the input control. Pierce™ Protein A/G Magnetic Beads (Thermo Fisher Scientific, Waltham, MA, USA) were prewashed and incubated with 5 µg of anti-m^6^A antibody or rabbit IgG for 2 h at 4 °C with rotation. After 3 washes, the antibody-conjugated beads were mixed with purified poly (A) RNA, and 1 × immunoprecipitation buffer supplemented with RNase inhibitors. Then, the methylated mRNAs were precipitated with 5 mg of glycogen and one-tenth volume of 3 M sodium acetate in a 2.5 volume of 100 % ethanol at − 80 °C overnight after proteinase K digestion. Further enrichment was calculated by qPCR along with the MeRIP RNAs using primers listed in Table S9. The relative enrichment of m^6^A in each sample was calculated by normalizing the Cq value of the m^6^A-IP portion to the corresponding input portion.

### Statistical analysis

All data (unless stated otherwise) are expressed as mean ± standard error of mean (SEM) and analyzed using GraphPad Prism software (v7.0). The comparisons of the Fs and Fts were made using the paired-samples design. For the results of RT-qPCR and WB, the statistical significance was calculated by using a paired *t*-test. Experiments were run in at least three independent replicates and differences were considered significant when *P* < 0.05.

## Supplementary Information

**Additional file 1: Table S1. ** Sequencing data for fresh and frozen-thawed boar sperm

**Additional file 2: Table S2-1. **Methylated RNA sites on mRNAs in fresh and frozen-thawed sperm.

**Additional file 3: Table S2-2.** Differentially methylated sites on mRNAs

**Additional file 4: Table S3.** Genes containing the top ten hyper-methylated peaks in boar Fts compared with Fs

**Additional file 5: Table S4.** Genes containing the top ten hypo-methylated peaks in boar Fts compared with Fs

**Additional file 6: Table S5-1.** GO analysis of up-methylated mRNAs

**Additional file 7: Table S5-2.** GO analysis of down-methylated mRNAs

**Additional file 8: Table S6-1.** KEGG analysis of up-methylated mRNAs

**Additional file 9: Table S6-2.** KEGG analysis of down-methylated mRNAs

**Additional file 10: Table S7-1.** mRNA expression profile of fresh and frozen-thawed sperm

**Additional file 11: Table S7-2.** Differentially expressed mRNAs in fresh and frozen-thawed sperm

**Additional file 12: Table S8.** Joint analysis of differentially methylated sites and differentially expressed mRNAs

**Additional file 13: Table S9.** Primers used for RT-qPCR

## Data Availability

All raw transcriptome data reported in this article have been deposited to the NCBI’s Gene Expression Omnibus, the web link is https://www.ncbi.nlm.nih.gov/geo/query/acc.cgi?acc=GSE164691.

## Abbreviations

PPP1R3B: Protein phosphatase 1 regulatory subunit 3B
NADK2: NAD kinase 2, mitochondrial
HIF1A: Hypoxia inducible factor 1 subunit alpha
MYD88: MYD88 innate immune signal transduction adaptor
SLC9A3R1: SLC9A3 regulator 1
FOXO3: Forkhead box O3
MCUR1: Mitochondrial calcium uniporter regulator 1
FASN: Fatty acid synthase
PKM: Pyruvate kinase, muscle
AMPK: Adenosine monophosphate-activated protein kinase
mTOR: Mammalian target of rapamycin
MAPK: Mitogen-activated protein kinases
