# Supplementary material for: Transcriptome-wide m6A profiling reveals mRNA post-transcriptional modification of boar sperm during cryopreservation
Source: BMC Genomics. 2021 Aug 3;22:588. doi: 10.1186/s12864-021-07904-8 (PMC8335898; doi:10.1186/s12864-021-07904-8)
Supplement: Supplementary file 1 — Additional file 1: Table S1. Sequencing data for fresh and frozen-thawed boar sperm [file 12864_2021_7904_MOESM1_ESM.docx]

**Table S1** Sequencing data for fresh and frozen-thawed boar sperm.

|  | **Samples ID** | **Raw reads** | **Clean reads** | **Mapped Reads** | **Mapping Ratio (%)** |
| --- | --- | --- | --- | --- | --- |
| IP-Seq | Fresh-1 | 84,990,906 | 84,647,836 | 68,813,865 | 81.29 |
|  | Fresh-2 | 88,256,904 | 87,902,468 | 71,028,485 | 80.8 |
|  | Fresh-3 | 84,907,708 | 84,564,310 | 68,263,686 | 80.72 |
|  | frozen-thawed-1 | 73,440,690 | 73,272,818 | 56,348,147 | 76.9 |
|  | frozen-thawed-2 | 85,535,666 | 85,330,238 | 64,826,909 | 75.97 |
|  | frozen-thawed-3 | 68,287,426 | 68,131,580 | 51,988,721 | 76.31 |
| Input-Seq | Fresh-1 | 94,515,420 | 92,334,406 | 70,708,492 | 76.58 |
|  | Fresh-2 | 84,442,320 | 82,320,922 | 61,812,064 | 75.09 |
|  | Fresh-3 | 75,679,080 | 74,308,144 | 58,186,020 | 78.3 |
|  | frozen-thawed-1 | 89,113,950 | 59,722,248 | 39,485,092 | 66.11 |
|  | frozen-thawed-2 | 85,546,736 | 66,020,988 | 45,103,118 | 68.32 |
|  | frozen-thawed-3 | 106,252,518 | 87,091,758 | 62,159,103 | 71.37 |
