# Supplementary material for: Transcriptome-wide m6A profiling reveals mRNA post-transcriptional modification of boar sperm during cryopreservation
Source: BMC Genomics. 2021 Aug 3;22:588. doi: 10.1186/s12864-021-07904-8 (PMC8335898; doi:10.1186/s12864-021-07904-8)
Supplement: Supplementary file 4 — Additional file 4: Table S3. Genes containing the top ten hyper-methylated peaks in boar Fts compared with Fs [file 12864_2021_7904_MOESM4_ESM.docx]

**Table S3** Genes containing the top ten hyper-methylated peaks in boar Fts compared with Fs.

| **Gene symbol** | **Chromosome** | **txStart** | **txEnd** | **Fold change** | **FDR** |
| --- | --- | --- | --- | --- | --- |
| ZBTB20 | 13 | 145,629,977 | 145,630,220 | 241.3 | 5.81E-04 |
| PCNX4 | 1 | 189,120,979 | 189,121,560 | 227.6 | 1.96E-03 |
| ENSSSCG00000031242 | 5 | 63,214,681 | 63,215,740 | 221.2 | 1.46E-04 |
| ENSSSCG00000033363 | 14 | 141,265,681 | 141,266,340 | 197 | 1.47E-04 |
| INTS11 | 6 | 63,598,035 | 63,598,390 | 182.4 | 2.97E-04 |
| ZNF786 | 9 | 109,249,181 | 109,249,960 | 176.7 | 2.40E-03 |
| ZHX1 | 4 | 16,124,784 | 16,125,080 | 168.2 | 2.74E-04 |
| RXFP1 | 8 | 47,601,981 | 47,602,880 | 165.9 | 1.66E-03 |
| PRPF3 | 4 | 98,742,141 | 98,742,820 | 163.7 | 2.85E-04 |
| XIRP2 | 15 | 73,733,201 | 73,733,588 | 160.8 | 3.43E-04 |

Note: txStart and txEnd represent the start/end position of the differentially methylated RNA peaks.
