# Supplementary material for: Transcriptome-wide m6A profiling reveals mRNA post-transcriptional modification of boar sperm during cryopreservation
Source: BMC Genomics. 2021 Aug 3;22:588. doi: 10.1186/s12864-021-07904-8 (PMC8335898; doi:10.1186/s12864-021-07904-8)
Supplement: Supplementary file 5 — Additional file 5: Table S4. Genes containing the top ten hypo-methylated peaks in boar Fts compared with Fs [file 12864_2021_7904_MOESM5_ESM.docx]

**Table S4** Genes containing the top ten hypo-methylated peaks in boar Fts compared with Fs.

| **Gene Name** | **chromosome** | **txStart** | **txEnd** | **Fold change** | **FDR** |
| --- | --- | --- | --- | --- | --- |
| B4GALNT4 | 2 | 200461 | 200900 | 461 | 2.29E-03 |
| ENSSSCG00000022794 | 7 | 120636121 | 120636521 | 450.3 | 1.42E-04 |
| ENSSSCG00000035683 | 9 | 2534781 | 2535140 | 409.3 | 1.42E-04 |
| FRS3 | 7 | 36960081 | 36960720 | 265.4 | 1.46E-04 |
| TAF5L | 14 | 60255071 | 60255460 | 242.2 | 1.21E-03 |
| PRSS21 | 3 | 39318401 | 39318640 | 195.5 | 1.42E-04 |
| TPRN | unknown | 710281 | 710720 | 191.9 | 2.22E-03 |
| CCDC22 | X | 43304401 | 43304840 | 189.7 | 1.42E-04 |
| CILP | 1 | 163247175 | 163247445 | 189.6 | 1.42E-04 |
| LRP11 | 1 | 16139101 | 16139460 | 186.2 | 2.37E-04 |

Note: txStart and txEnd represent the start/end position of the differentially methylated RNA peaks.
