# Supplementary material for: Transcriptome-wide m6A profiling reveals mRNA post-transcriptional modification of boar sperm during cryopreservation
Source: BMC Genomics. 2021 Aug 3;22:588. doi: 10.1186/s12864-021-07904-8 (PMC8335898; doi:10.1186/s12864-021-07904-8)
Supplement: Supplementary file 13 — Additional file 13: Table S9. Primers used for RT-qPCR [file 12864_2021_7904_MOESM13_ESM.docx]

**Table S9** Primers used for RT-qPCR.

| **Gene symbol** | **Primer sequence (5'→3')** | **Product length (bp)** | **GenBank accession** |
| --- | --- | --- | --- |
| *GAPDH* | F: ACTCACTCTTCTACCTTTGATGCT | 100 | NM_001206359.1 |
|  | R: TGTTGCTGTAGCCAAATTCA |  |  |
| *METTL3* | F: CCCTATGGGACCCTGACAGA | 145 | XM_003128580.5 |
|  | R: CTACCCGTTCGTAACCCCAG |  |  |
| *METTL14* | F: GCATCCTACTTCAGTGCCCC | 172 | XM_003129231.6 |
|  | R: ATCGATTTCTTTCCCGCCCA |  |  |
| *ALKBH5* | F: CGCAGAGCTGTCATCATCCT | 118 | XM_021067995.1 |
|  | R: TGTTTCCCGACAGACGATCC |  |  |
| *FTO* | F: TTTGGCGATACCCCTTCACC | 149 | NM_001112692.1 |
|  | R: TGTAATCCAAGGCTCCCGTC |  |  |
| *YTHDF2* | F: AACAAGGGTCCTGTGGCAAA | 148 | XM_005665152.3 |
|  | R: GCTGTGTCTGTTGCCCTACT |  |  |
| *PPP1R3B* | F: CGAAATCCAGCACGAAGGTC  R: AGAAGCGGGTGTCCTTTGC | 165 | XM_005671823.3 |
| *NADK2* | F: CCAGCTTTTGCCAGTGAGAG  R: GGAAGCCAGTCCCTTTAGAGT | 133 | XM_003359754.4 |
| *HIF1A* | F: TGGTACTCACAGATGATGGTGAC  R: CATTTCCTCATGGTCGCACG | 131 | NM_001123124.1 |
| *SLC9A3R1* | F: GACAGCCCCAAGAAAGAGGAC  R: CGGCCAGGGAGATGTTGA | 85 | NM_001143724.1 |
| *FOXO3* | F: GGGGAGTTTGGTCAATCAGA  R: TGCATAGACTGGCTGACAGG | 168 | NM_001135959.1 |
| *MYD88* | F: GCTGGAACAGACCAACTAT  R: TCCTTGCTTTGCAGGTAAT | 153 | NM_001099923.1 |
| *MCUR1* | F: AGTCAAAGAATTGCACGCCC  R: TCCACAGGCGGTAAAATCCC | 179 | XM_005665577.3 |
| *FASN* | F: CCCGAATCTGCACTACCACA  R: AGCCGAAGGAGTTTATGCCC | 122 | NM_001099930.1 |
| *PKM* | F: GCGGCAGCTTTGATAGTTCTG  R: TCCTGCACTGGGTCCTTACA | 167 | XM_021099115.1 |
| *ACLY* | F: CACCCCTCTGCTTGACTACG  R: AGGACCCGCAGTTTCTAAGC | 128 | NM_001105302.1 |
